# Chronotype Predicts Body Mass Index via Emotion Regulation Strategy Use and Emotional Eating

**DOI:** 10.1002/brb3.70542

**Published:** 2025-05-08

**Authors:** Gregory S. Keenan, Sakina Hosseni, Robert C. A. Bendall

**Affiliations:** ^1^ School of Psychology Liverpool John Moores University Liverpool UK; ^2^ School of Health and Society University of Salford Salford UK; ^3^ Centre for Applied Health Research University of Salford Salford UK

**Keywords:** body mass index, chronotype, cognitive reappraisal, emotion regulation, emotional eating, expressive suppression, morningness‐eveningness

## Abstract

**Introduction:**

Rates of obesity are increasing across all regions representing a critical public health concern. An evening chronotype has been associated with elevated body mass index and a less nutritious diet. However, the mechanisms underpinning the relationship between chronotype and body mass index remain unclear. The aim of the current study was to investigate if chronotype is indirectly associated with body mass index via emotion regulation strategy use and emotional eating.

**Method:**

Participants completed the Morningness‐Eveningness Questionnaire to assess chronotype, the Emotion Regulation Questionnaire to assess habitual emotion regulation strategy use, the Warwick‐Edinburgh Mental Well‐being Scale to assess mental well‐being, and the Three‐Factor Eating Questionnaire to provide a measure of emotional eating. Participants reported their weight and height to allow body mass index to be calculated. Structural equation modeling tested the predicted indirect association between chronotype and body mass index via emotion regulation strategy use and emotional eating.

**Results:**

Chronotype was indirectly associated with body mass index via emotion regulation strategy use and emotional eating. As predicted, individuals with an evening chronotype tended to report greater use of expressive suppression, which was associated with a greater tendency to emotionally eat and a higher body mass index (*p* = 0.008). In contrast, individuals with a morning chronotype reported more frequent use of cognitive reappraisal, which was associated with reduced emotional eating and a lower body mass index (*p* = 0.003). The direct pathway between chronotype and body mass index was non‐significant (*p* = 0.821).

**Conclusion:**

These findings suggest a clear pathway through which chronotype might be associated with body mass index, with evening chronotypes at a greater risk of weight gain. Our results suggest that it is not the independent influence of emotion regulation strategy use *or* emotional eating on its own that is important in the association between chronotype and body mass index, but the *combined sequential effect* of a general tendency towards an emotion regulation strategy and then the impact this has upon emotional eating that is important. The findings highlight the importance of considering emotion regulation strategy use and emotional eating when designing interventions or therapies aimed at reducing obesity.

## Graphical abstract

Individuals with an evening chronotype reported greater use of expressive suppression, which was associated with a greater tendency to emotionally eat and a higher body mass index (BMI). In contrast, individuals with a morning chronotype reported more frequent use of cognitive reappraisal, which was associated with reduced emotional eating and a lower BMI. These findings suggest a clear pathway through which chronotype might be associated with BMI, with evening chronotypes at a greater risk of weight gain.

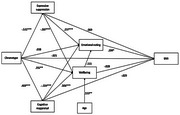

## 1 Introduction

As of recent estimates approximately 890 million adults globally are living with obesity (World Health Organization 2024). Rates of obesity are steadily increasing across all regions, and if current trends prevail, projections are that 51% of the world's adult population (nearly two billion) could be living with overweight or obesity by the year 2035 (World Obesity Federation 2024). This represents a major public health concern due to the elevated risk of developing cardiovascular disease, type 2 diabetes, certain types of cancer, and various mental health conditions (Berrington de Gonzalez et al. 2010; Leutner et al. 2023; Scully et al. 2021; Wormser et al. 2011). Identifying key determinants of weight gain and identifying those at greatest risk of weight gain are therefore of importance in the development of effective interventions and countermeasures.

The causes of obesity are complex, but one factor that has been associated with weight gain is chronotype (Lucassen et al. 2013; Teixeira et al. 2022; van der Merwe et al. 2022; Yu et al. 2015). Chronotype refers to an individual's natural preference to sleep at a certain time and is the behavioral manifestation of our circadian rhythm (Salehinejad et al. 2021). Chronotypes are often categorized into morning and evening chronotypes, with a morning (or “lark”) chronotype characterized by feeling most awake and alert in the early part of the day and preferring to go to bed early. In contrast, an evening (or “night owl”) chronotype is associated with feeling more active and alert late in the day or at night and preferring to stay up later into the evening.

Research investigating the associations between chronotype and body mass index (BMI) has historically focused on physiological mechanisms. For example, it is well established that evening chronotypes generally engage in lower levels of physical activity (Sempere‐Rubio et al. 2022), are more prone to irregular eating patterns (Teixeira et al. 2022), and are more likely to consume high‐calorie, low‐nutrient foods, including sweetened beverages and fast foods (Gontijo et al. 2019; Li et al. 2018). Evening chronotypes also often experience shorter sleep duration and poorer sleep quality (Yu et al. 2015), with sleep deprivation known to increase levels of ghrelin and inhibit leptin secretion, promoting excess energy intake (Taheri et al. 2004).

Growing evidence, however, also points towards the role of psychological mechanisms in the association between chronotype and obesity. For example, it is well established that individuals with an evening chronotype are more prone to experiencing lower levels of well‐being, as well as greater chances of depression and anxiety (Antypa et al. 2016; Au and Reece 2017; Levandovski et al. 2011; Merikanto et al. 2013; Norbury 2021). This has been shown with large population cohort studies (Antypa et al. 2016), in longitudinal data sets (Druiven et al. 2020), and in studies using ecological momentary assessments (Hasler et al. 2012). It is also argued that evening chronotypes may be more susceptible to reduced well‐being due to their likelihood of having a delayed and blunted positive affect rhythm (Miller et al. 2015), but also due to reduced sleep quality and duration, especially in individuals obliged to wake earlier than desired due to work or social commitments (Dmitrzak‐Węglarz et al. 2016; Juda et al. 2013; Lima et al. 2010; Roeser et al. 2012).

Recently, evidence has begun to emerge suggesting that chronotype might also be associated with emotion regulation processes. Watts and Norbury (2017) found that an evening chronotype was associated with an increased tendency to habitually adopt a suppression‐based emotion regulation strategy, whereby individuals seek to deal with emotions through avoidance or by trying to ignore them, often by inhibiting ongoing emotion‐expressive behavior (Gross and John 2003). While adopting expressive suppression may have short‐term benefits and assist in reducing or inhibiting emotional expression of an emotional experience, this strategy is often described as being maladaptive, as long‐term or chronic use is less effective at reducing emotional and physiological arousal (Gross and John 2003). For example, using expressive suppression has been shown to predict negative affect and diminished well‐being in both clinical and non‐clinical populations (Aldao et al. 2010; Bendall et al. 2024; Campbell‐Sills et al. 2006; Cisler et al. 2010; Gotlib and Joormann 2010; Kraiss et al. 2020; Latif et al. 2019; Mennin et al. 2003; Tsypes et al. 2013). In contrast, a morning chronotype has been shown to be associated with increased use of a different emotion regulation strategy: cognitive reappraisal (Watts and Norbury 2017), which is characterized by the generation of positive interpretations of stressful events to reduce distress (Gross and John 2003). Cognitive reappraisal is often regarded as a positive emotion regulation strategy, associated with beneficial well‐being outcomes (Aldao et al. 2010, Aldao et al. 2014; Gross and John 2003; McMahon and Naragon‐Gainey 2018a, 2018b).

Another psychological variable linking chronotype to weight gain is emotional eating, whereby individuals consume food in response to negative feelings rather than hunger or satiety cues (e.g., Konttinen et al. 2014; Merikanto et al. 2013; Vera et al. 2018). In particular, it appears that those with an evening chronotype might be more prone to emotional eating (Konttinen et al. 2014; Merikanto et al. 2013; Vera et al. 2018). Of relevance to the current study, emotion regulation strategy also appears to be associated with emotional eating, with those who use an emotional suppression strategy more likely to consume food in response to negative emotions (Evers et al. 2010; Ferrer et al. 2017). Furthermore, recent work by Herren et al. (2021) has indicated that an emotional suppression strategy might predict BMI via a pathway involving emotional eating, such that those individuals who suppress negative emotions are more likely to eat in response to these emotions, with the consequence that they develop higher BMI scores.

How chronotype, emotion regulation, emotional eating, and well‐being might relate to one another and whether they combine to explain the relationship between chronotype and weight gain is not fully clear. Considering the literature reviewed above, there appears to be evidence for direct links between many of these variables (e.g., a morning chronotype and use of cognitive reappraisal; an evening chronotype and use of expressive suppression; emotion regulation strategy (cognitive reappraisal or expressive suppression) and both well‐being and reduced emotional eating; emotional eating and higher BMI). The novel contribution of the current study is in combining these individual associations and testing whether a serial relationship might exist between chronotype and BMI via emotion regulation strategy use (expressive suppression or cognitive reappraisal) and emotional eating. To the best of our knowledge, this pathway has not yet been formally tested. Two predictions were subsequently made:

H1: Individuals with an evening chronotype would be more likely to use expressive suppression, which in turn would be associated with increased emotional eating and subsequently a higher BMI.

H2: Individuals with a morning chronotype would be more likely to use cognitive reappraisal, which in turn would be associated with reduced emotional eating and subsequently a reduced BMI.

Well‐being was also measured so we could establish if this played a role in either of the two hypothesized pathways. For example, it could be that a pathway only exists between expressive suppression and emotional eating when well‐being is included.

## 2 Methods

### 2.1 Participants

Participants were recruited online via an opportunity sample of staff and students at the University of Salford and from members of the public via adverts on social media. Student participants recruited via the university did so in exchange for course participation credits. Non‐student participants did not receive any incentives or payment. Exclusion criteria included a past or current diagnosis of an eating disorder. Based on the formula by Kim (2005), it was estimated that a minimum sample of 183 would be needed to achieve a close‐fitting root‐mean‐square error of approximation (RMSEA) (90% power at alpha = 0.05, *df* = 24). The final sample included 202 participants between the ages of 18 and 55 (*M* = 23.62, *SD* = 5.28), consisting of 148 females and 54 males. The School of Health Sciences and School of Health and Society Ethics Committee at the University of Salford provided ethical approval for the current study (approval number: 4724).

### 2.2 Measures

Researchers prepared questions and collected participants age (in years) and gender (female, male, non‐binary, other, with an option to provide more details if they selected other). Participants provided self‐reported height (in centimeters) and weight (in kilograms) to calculate BMI (kg/m^2^). Self‐reported BMI has previously been shown to correlate well with objectively measured BMI (Ng et al. 2011; Pursey et al. 2014).

The Morningness‐Eveningness Questionnaire (MEQ; Horne and Östberg, 1977) was administered to assess circadian preference. The MEQ is a validated 19‐item scale, with individuals responding on different 4‐ or 5‐point Likert scales. An example question is, “At approximately what time in the evening do you feel tired and, as a result, in need of sleep?” Scores are summed with a range of between 16 and 86. Scores below 41 are indicative of “evening types,” between 42 and 58 are indicative of “intermediate types,” and values of 59 and higher are indicative of “morning types”. Cronbach's Alpha (*α*) values for the scale were 0.917.

The Emotion Regulation Questionnaire (ERQ; Gross and John 2003) was used to record habitual use of two emotion regulation strategies. The ERQ is a validated 10 item scale, with six questions that measure tendencies to use cognitive reappraisal (e.g., “I control my emotions by changing the way I think about the situation I'm in”) and four questions that assess habitual use of expressive suppression (e.g., “I keep my emotions to myself”). Responses are recorded using a 7‐point Likert scale (response options: 1 = strongly disagree, 4 = neutral, 7 = strongly disagree). The minimum and maximum scores for cognitive reappraisal are 6 and 42, with the minimum and maximum scores for expressive suppression 4 and 28, respectively. A higher score on either subscale indicates a greater tendency to use that particular emotion regulation strategy. Cronbach's alpha values for two subscales were cognitive reappraisal *α* = 0.928 and expressive suppression *α* = 0.843.

Participants completed the Warwick–Edinburgh Mental Well‐being Scale (WEMHWS; Tennant et al. 2007) to provide a measure of well‐being. The WEMHWS is a validated 14‐item scale that asks individuals about their current thoughts and feelings regarding themselves and their outlook (e.g., “I've been feeling confident”; response options: 1 = none of the time, 2 = rarely, 3 = some of the time, 4 = often, 5 = all of the time). Responses are summed, yielding a minimum possible score of 14 and a maximum possible score of 70. Higher scores represent a better sense of well‐being. In the current study population, *α* = 0.934.

The 18‐item validated Three‐Factor Eating Questionnaire (TFEQ; Karlsson et al. 2000) was used to provide a measure of emotional eating. The TFEQ assesses three different aspects of eating behavior, including emotional eating, cognitive restraint, and uncontrollable eating. For the current study, only the emotional eating questions were of interest. Three items measured emotional eating (e.g., “When I feel anxious, I find myself eating”) on a 4‐point Likert scale (4 = definitely true, 3 = mostly true, 2 = mostly false, 1 = definitely false). The emotional eating subscale has a minimum possible score of 4 and a maximum possible score of 12. A higher score represents greater levels of emotional eating. In the current study population, *α* = 0.824.

### 2.3 Procedure

Questionnaires were hosted on JISC online Surveys (Bristol, UK). Participants recruited via advertisements on social media and via the University of Salford student recruitment site accessed the questionnaire via a web link. Participants first read an information sheet before providing informed consent and then demographic information (including height and weight). To ensure consistency across participants, the following questionnaires were then presented in a fixed order: MEQ, ERQ, WEMWBS, and TFEQ. Participants were informed that they could withdraw from the study at any time without consequences.

### 2.4 Data Analyses

The data from the current study are available in the Open Science Framework repository (https://osf.io/xm3ea/)^1^. A structural equation model was generated to test the hypotheses that individuals with an evening chronotype would be more likely to use expressive suppression, which in turn would be associated with increased emotional eating and subsequently a higher BMI, and second, that individuals with a morning chronotype would be associated with increased use of cognitive reappraisal, which in turn would be associated with reduced emotional eating and a subsequently reduced BMI. Data modeling was conducted in AMOS version 28 (IBM, New York). In total, 210 participants reached the end of the survey, but only 202 provided complete responses, which are needed to calculate bootstrapped indirect effects. Of those removed, four participants provided implausible age values (age < 5 or > 120), one provided incomplete height and weight (necessary to calculate BMI), and three provided BMI values that appeared to be inaccurate (< 10 or > 66). These were removed as complete responses are needed to calculate bootstrapped indirect effects.

Model fit was assessed through a variety of different indices. Scores under 0.08 were deemed to represent a good fit for the standardized root mean residual (SRMR). Scores under 0.06 were deemed to represent a good fit for the root mean square error of approximation (RMSEA) parsimony adjusted measure, and values greater than 0.06 but less than 0.08 were deemed acceptable (Hu and Bentler 1998). Values of 0.95 were considered to represent good fit for the Tucker–Lewis index (TLI) and Comparative Fit Index (CFI), and values above 0.90 as acceptable (Hu and Bentler 1998).

**FIGURE 1:**
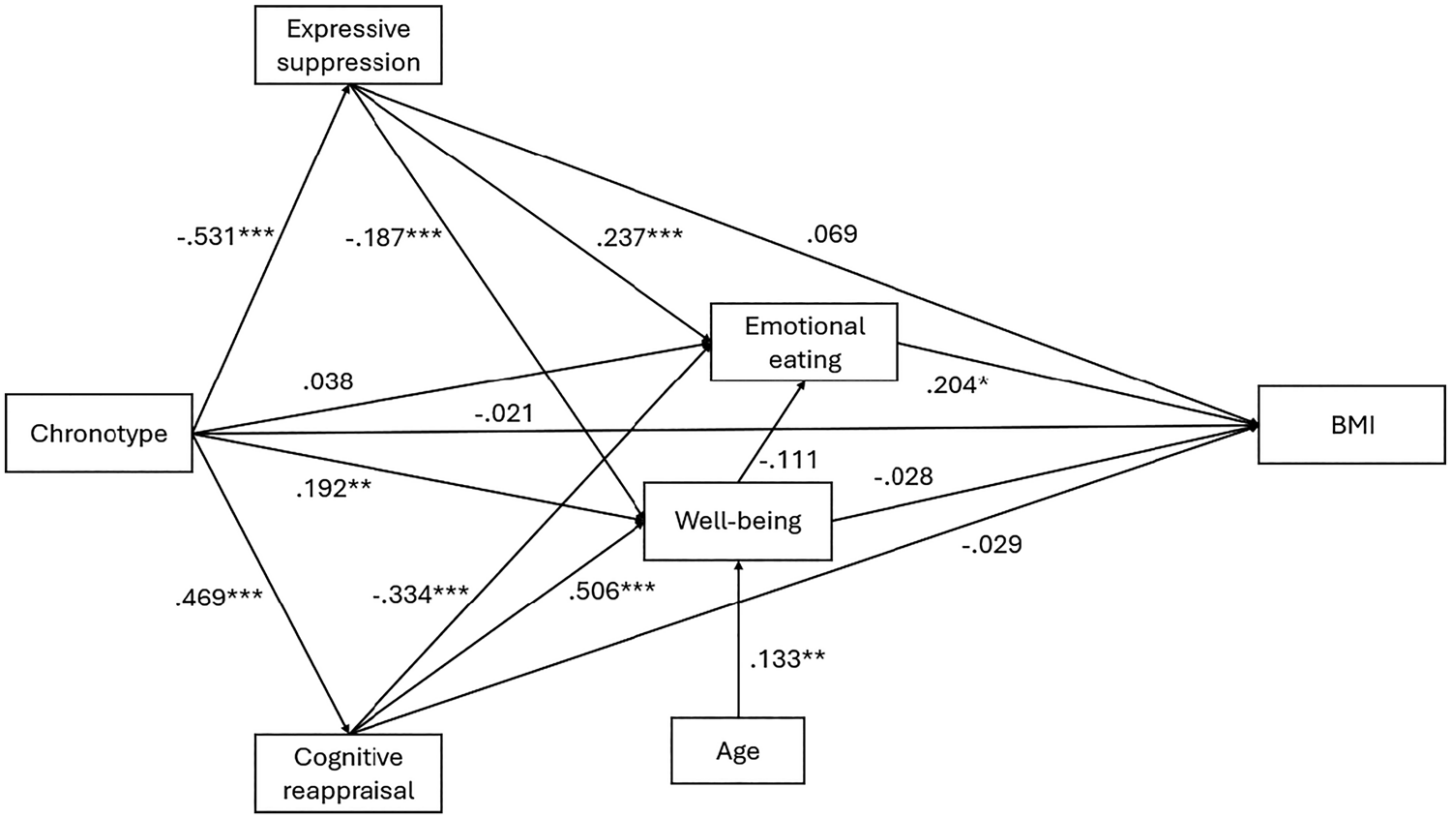
Associations between chronotype, emotion regulation strategy (expressive suppression vs. cognitive reappraisal), well‐being, emotional eating, and BMI. Standardized regression coefficients are reported: *** *p* < 0.001, * *p* < 0.005, ** *p* < 0.01. To aid interpretation, residuals and covariances are not visually represented.

The hypothesized indirect effects between chronotype and BMI were assessed via bias‐corrected bootstrapping using 95% confidence intervals (N = 1000). Direct effects between variables are reported as standardized regression coefficients in Figure 1 and as unstandardized regression coefficients in Table 2.

Before building the model, the effect of age on each variable in the model was explored via correlations. Where age had a statistically significant influence, this relationship was included in the model.

## 3 Results

### 3.1 Descriptive Statistics

The sample (*N* = 202) was mostly female (73.3%), with 15.4% identifying as having a morning chronotype, 46.0% as intermediate, and 38.6% as evening chronotypes. Within the sample, 5.0% were underweight, 57.4% were of healthy weight, 26.2% were overweight, and 11.4 % were living with obesity. The means and standard deviations for variables included within the model are presented in Table 1.

**TABLE 1:** Sample descriptives and questionnaire scores (N = 202).

	Mean	Standard deviation	Range of scores
Chronotype ^a^	46.47	12.93	21–85
Expressive suppression ^b^	16.51	6.37	4–28
Cognitive reappraisal ^b^	26.47	9.21	9 42
Well‐being ^c^	43.23	12.01	14–68
Emotional eating ^b^	7.30	2.56	3–12
Body mass index (kg/m^2^)	24.23	5.28	16.3–49.2
Age (in years)	23.62	5.94	18–55

^a^ Higher scores represent a greater tendency towards a morning chronotype; a lower score, towards an evening chronotype.

^b^ Higher scores represent a greater tendency towards these traits, e.g., expressive suppression, cognitive reappraisal, and emotional eating.

^c^ Higher scores represent better self‐reported mental well‐being.

### 3.2 Model Evaluation

The final model with covariances included was a very good fit for the data (CFI = 0.997, TLI = 0.981, SRMR = 0.024, RMSEA = 0.041). A covariance was added between the error terms for the two emotional regulation strategies (expressive suppression and cognitive reappraisal), the error term for BMI and age, and between age and chronotype. Covariances were added based on their theoretical rationale and the necessity for these to exist between exogenous variables (i.e., age and chronotype).

### 3.3 Pathways Between Chronotype and BMI

It was hypothesized that chronotype would be indirectly associated with BMI via emotion regulation strategy (emotional suppression and cognitive reappraisal) and emotional eating. Consistent with this prediction, there was no direct association between chronotype and BMI (Figure 1, see Table 2 for direct effects), but there was a significant indirect effect via expressive suppression and emotional eating and another significant indirect effect via cognitive reappraisal and emotional eating (see Table 3 for indirect associations). As such, having an evening chronotype was directly associated with a greater self‐reported tendency to engage in expressive suppression, with expressive suppression then positively associated with emotional eating, and emotional eating with increased BMI. In contrast, a morning chronotype was associated with a greater tendency to engage in cognitive reappraisal, which was then associated with a reduced tendency to emotionally eat and a lower BMI.

**TABLE 2:** Direct unstandardized regression coefficients between variables.

Association	b (Standard error)	*p*	95%I
Chronotype → expressive suppression	−0.261 (0.029)	< 0.001	−0.303–−0.216
Chronotype → cognitive reappraisal	0.333 (0.044)	< 0.001	0.270–0.398
Chronotype → well‐being	0.179 (0.055)	0.001	0.075–0.305
Chronotype → emotional eating	0.007 (0.015)	0.626	−0.016–0.031
Chronotype → BMI	−0.008 (0.035)	0.821	−0.071–0.064
Expressive suppression → well‐being	−0.355 (0.106)	< 0.001	−0.546–−0.143
Expressive suppression → emotional eating	0.095 (0.029)	0.001	0.041–0.149
Expressive suppression → BMI	0.057 (0.069)	0.411	−0.078–0.193
Cognitive reappraisal → well‐being	0.663 (0.070)	< 0.001	0.498–0.812
Cognitive reappraisal → emotional eating	−0.093 (.023)	< 0.001	−0.129–−0.057
Cognitive reappraisal → BMI	−0.016 (0.055)	0.763	−0.092–0.063
Well‐being → emotional eating	−0.024 (0.019)	0.206	−0.056–0.014
Well‐being → BMI	−0.012 (0.044)	0.777	−0.080–0.038
Emotional eating → BMI	0.420 (0.163)	0.010	0.181–0.653
Age → well‐being	0.271 (0.096)	0.005	0.018–0.476

**TABLE 3:** Hypothesized indirect effects.

Association	b (Standard error)	*p*	95% CI
Chronotype → expressive suppression → emotional eating → BMI	−0.010 (0.006)	0.008	−0.023–−0.003
Chronotype → expressive suppression → well‐being → emotional eating → BMI	−0.001 (0.001)	0.142	−0.010–0.019
Chronotype → expressive suppression → BMI	−0.015 (0.021)	0.448	−0.056–0.004
Chronotype → cognitive reappraisal → emotional eating → BMI	−0.013 (0.006)	0.003	−0.027–−0.006
Chronotype → cognitive reappraisal → well‐being → emotional eating → BMI	−0.002 (0.003)	0.252	−0.006–0.001
Chronotype → cognitive reappraisal → BMI	−0.003 (0.015)	0.903	−0.034–0.021
Chronotype → well‐being → emotional eating → BMI	−0.002 (0.002)	0.251	−0.006–0.001
Chronotype → emotional eating → BMI	0.003 (0.007)	0.506	−0.004–0.022

* *Note*: indirect effects are calculated by multiplying the different sub‐components of the indirect pathway. If any of these components are negative, this will result in a negative indirect effect.

In the current data, both emotion regulation strategy and emotional eating are essential in accounting for the indirect associations between chronotype and BMI. As can be seen in Table 3, where only the emotion regulation strategy is included as a mediator (and emotional eating is omitted), the association between chronotype and BMI is non‐significant. Similarly, where only emotional eating is considered as a mediator (and either emotion regulation strategy is omitted), this pathway is non‐significant. Both the emotion regulation strategy and emotional eating were therefore necessary for a significant pathway to be observed. It is also worth noting that while chronotype and the two emotion regulation strategies were directly associated with well‐being, well‐being was not a mediator of the relationship between chronotype and BMI.

## 4 Discussion

The current study sought to establish whether emotional eating and two emotion regulation strategies– expressive suppression and cognitive reappraisal– might explain the association between chronotype and BMI. As predicted, individuals with an evening chronotype reported being more likely to use expressive suppression, which in turn was associated with a greater tendency to emotionally eat and to then have a higher BMI. In stark contrast, individuals with a morning chronotype were more likely to use cognitive reappraisal as an emotion regulation strategy. Using cognitive reappraisal was then associated with a reduced tendency to emotionally eat, and these individuals subsequently reported a lower BMI. Interestingly, the direct path between chronotype and BMI was non‐significant, as were the pathways between chronotype to emotion regulation and then BMI as well as between chronotype to emotional eating and then BMI. Crucially, this suggests that it is not simply the emotion regulation strategy *or* emotional eating on its own that might be important in the association between chronotype and BMI, but the *combined sequential effect* of a general tendency towards an emotion regulation strategy and then the impact this has upon emotional eating that might be key.

These novel results indicate a clear pathway through which chronotype is associated with BMI, with evening chronotypes at a greater risk of weight gain. Indeed, our findings suggest modifiable psychological constructs that could be therapeutically targeted to prevent weight gain and obesity in individuals with an evening chronotype. There has been a large volume of research on how to reduce emotional eating (Chew et al. 2022; Lattimore 2020; Smith et al. 2023), but the current data indicate that targeting the emotion regulation strategy people use might be a novel treatment avenue. For example, educational resources for individuals with an evening chronotype about the risks of expressive suppression and the link to emotional eating could be a preventative measure. Conversely, teaching people with an evening chronotype how to implement cognitive reappraisal emotion regulation techniques could reduce the likelihood of engaging in emotional eating. As an indicator of the potential effectiveness of targeting emotion regulation strategies, studies have shown that emotion regulation therapy can reduce symptom severity in generalized anxiety disorder and major depressive disorder (Renna et al. 2018; Scult et al. 2019). If treatment can be effective with these disorders, targeting emotion regulation strategy use may be effective in limiting the likelihood that individuals eat to alleviate negative affect. Further work is ultimately needed to investigate if targeting emotion regulation strategy use can help drive improvements in eating behavior and obesity.

There is a somewhat limited literature investigating the associations between chronotype and emotion regulation, making replication of these initial findings important. Our finding that those with an evening chronotype reported being more likely to use expressive suppression, while those with a morning chronotype reported being more likely to use cognitive reappraisal, is in line with previous findings (Watts and Norbury 2017). However, other research has suggested that cognitive reappraisal, but not expressive suppression, is associated with chronotype, whereby individuals with a morning chronotype more frequently adopted cognitive reappraisal compared to individuals with an evening chronotype (Antúnez 2020). Related research has shown that expressive suppression moderates the relationship between chronotype and recognition of sadness and anger, whereby chronotype predicted emotion recognition response times only for individuals with high levels of expressive suppression (Santos et al. 2023). This research showing individuals with an evening chronotype have a bias for negative information is supported in other studies assessing emotional biases in evening chronotypes (Horne and Östberg, 1977; Lunn and Chen 2022). Our novel findings extend our understanding, demonstrating that chronotype is related to BMI via associations in emotional processing, including emotion regulation strategy use and emotional eating.

The current work furthers our understanding regarding the association between chronotype and BMI in a non‐clinical sample and provides the basis for research in clinical populations such as those with obesity or eating disorders. However, further investigation is needed on certain components of the model. While cognitive reappraisal and expressive suppression are the most studied emotion regulation strategies, alternative strategies may also help to explain the chronotype‐BMI association. For instance, problem‐solving, acceptance, and rumination are examples of additional emotion regulation strategies that are used to influence the duration and intensity of negative emotions (Young et al. 2019), and these could be associated with eating behaviors. Further, it has been suggested that flexible use of emotion regulation strategies i.e., inter‐ and intra‐individual variability in the adaptive use of emotion regulation strategies is key to successful emotion regulation and well‐being (Aldao et al. 2015; Bonanno and Burton 2013). Future research is needed to investigate if and how the flexible use of emotion regulation strategies across different contextual and situational environments is associated with emotional eating. The finding that emotional eating (and emotion regulation) is associated with the relationship between chronotype and BMI is supported by recent research from the sleep quality literature. Here, poor sleep quality and emotional eating were associated with higher BMI levels, with emotional eating mediating the influence of sleep quality on BMI (Zerón‐Rugerio et al. 2022). Further research could test (1) if emotion regulation strategy use is also able to help explain the sleep quality‐emotional eating‐BMI pathway identified by Zerón‐Rugerio et al. (2022) and (2) assess the individual contributions of chronotype and sleep quality on BMI (via emotion regulation and emotional eating).

A number of limitations exist with the current study. For instance, the findings are cross‐sectional and are therefore not causal. Research adopting experimental and longitudinal designs, as well as research testing the efficacy of interventions, would be needed to provide causal inferences on the novel observations reported here. Additionally, it is possible that other psychological variables that were not measured in the current study could be involved in the chronotype‐BMI association. The study also utilized a validated self‐report measure to assess chronotype, the MEQ, rather than adopting more direct measures such as sleep‐wake actigraphy or genetic profiles. However, self‐reported circadian preference as measured with the MEQ is correlated with genetic factors associated with chronotype variability and circadian preference measured via actigraphy (Roveda et al. 2017; Schneider et al. 2022; Vera et al. 2018). Additionally, the current work was not intended to assess whether the associations between chronotype and BMI were influenced by gender, and this is a potential avenue for further research.

To conclude, adopting structural equation modeling, we provide novel evidence of a pathway showing a route by which chronotype is associated with BMI. Specifically, those with an evening chronotype reported being more likely to use expressive suppression, which in turn predicted increased emotional eating and subsequently greater BMI scores. In contrast, those with a morning chronotype reported being more likely to use cognitive reappraisal, which predicted reduced emotional eating and subsequently lower BMI scores. These findings demonstrate that within our study population, emotion regulation, and emotional eating are essential in explaining the chronotype‐BMI association, suggesting that individuals with an evening chronotype are more at risk of weight gain. Interventions or prevention measures that seek to reduce obesity or BMI by accounting for an individual's chronotype should simultaneously consider targeting emotion regulation and emotional eating strategy use, especially for individuals with an evening chronotype.

## Author Contributions

**Gregory S. Keenan**: formal analysis, methodology, software, validation, visualization, writing – original draft, writing – review and editing. **Sakina Hosseni**: conceptualization, investigation, methodology, resources, software. **Robert C. A. Bendall**: conceptualization, data curation, methodology, project administration, resources, supervision, validation, writing – original draft, writing – review and editing.

## Ethics Statement

Ethical approval was obtained from the School of Health Sciences and School of Health & Society Ethics Committee at University of Salford.

## Consent

Informed consent was obtained from each participant. All methods were carried out in accordance with the relevant guidelines and regulations.

## Conflicts of Interest

The authors declare no conflicts of interest.

### Peer Review

The peer review history for this article is available at https://publons.com/publon/10.1002/brb3.70542.

## Data Availability

The datasets analyzed during the current study are available in the Open Science Framework repository (https://osf.io/xm3ea/).

## Endnotes

1 The current data were presented at the British Feeding and Drinking Group Annual Conference 2023 (Bendall and Keenan, 2023).
